# Evaluation of the effectiveness of some essential oils against zoonotic methicillin-resistant *Staphylococcus aureus* isolated from dairy products and humans

**DOI:** 10.5455/javar.2024.k778

**Published:** 2024-06-08

**Authors:** Marwa B. Salman, Asmaa Ibrahim Abdelaziz Zin Eldin, Nourhan Eissa, Ahmed Maher, Abd-Elghany Aish, Sherein I. Abd El-Moez

**Affiliations:** 1 Department of Zoonotic Diseases, National Research Center (NRC), Cairo, Egypt; 2 Department of Microbiology and Immunology, National Research Center (NRC), Cairo, Egypt; 3 Department of Animal Hygiene and Zoonoses, Faculty of Veterinary Medicine, University of Sadat City, Menoufia, Egypt; 4 Department of Hepatogastroenterology and Infectious Diseases, Shebin-Elkom Teaching Hospitals, Menoufia University, Menoufia, Egypt

**Keywords:** Egypt, Essential oils, MDR, MRSA, *S.*aureus

## Abstract

**Objective:**

*Staphylococcus aureus* (*S. aureus*) is a zooanthroponotic, nosocomial, and community-associated pathogen that threatens livestock management and even public health. The goal of this investigation was to clarify the role of *S. aureus* in zoonotic illnesses. Besides that, a novel trial was conducted in the current Egyptian study using oil extracts such as cactus oil, tea oil, geranium oil, and thyme oil to demonstrate the susceptibility of methicillin-resistant *S. aureus* (MRSA) isolates to these organic oils in response to the alarming global concern regarding the decreased susceptibility of *S. aureus* to known antibiotics, which exacerbates control and treatment protocols.

**Materials and Methods:**

A total of 110 samples (45 raw cattle milk samples, 35 Karish cheese samples, and 30 human sputum samples) were collected. The bacterium was identified via traditional culturing methods, Gram staining, and the application of several biochemical tests. After that, various kinds of known commercial antibiotics were used to detect the antimicrobial susceptibility (AMS) of the obtained isolates. Furthermore, conventional polymerase chain reaction (PCR) testing was performed to identify *S. aureus* (*nuc *gene) and MRSA (*mec*A gene), with further application of multiplex PCR for screening of all the obtained isolates for vancomycin resistance via targeting *van*A, *van*B, and *van*C genes. Finally, the agar gel diffusion method was performed to assess the antibacterial activity of four plant extracts (cactus oil, tea oil, geranium oil, and thyme oil) against the obtained MRSA.

**Results:**

The culturing method revealed *S. aureus* positivity in raw cattle milk (13.33%), in Karish cheese (28.57%), and in human samples (20%). The obtained isolates showed mainly resistance to amoxicillin-clavulanic and ampicillin antibiotics, while the dairy samples showed further resistance against ceptaxime and an intermediate reaction against erythromycin. On the molecular side, PCR positivity was present in human samples (10%), raw cow milk (13.33%), and Karish cheese (14.29%). Nine of the fourteen PCR isolates were methicillin-resistant *S. aureus* (MRSA) isolates. Comparing the four oil extracts against the acquired MRSA isolates, cactus oil extract proved to be the most effective.

**Conclusion:**

The study’s results are highly promising as they support the notion that certain essential oils possess strong antimicrobial properties against zoonotic *S. aureus*, thereby reducing the excessive use of antibiotics in veterinary and medical settings.

## Introduction

Among both animal and human populations, *Staphylococcus aureus* (*S. aureus*) is a well-known zooanthroponotic, invasive nosocomial, community-associated, and livestock-associated pathogen [1]. According to Rodrigues et al. [2], the pathogenicity of that Gram-positive pathogen is often mediated by certain virulence factors known as staphylococcal superantigens, which are responsible for clinical or even subclinical mastitis among infected dairy cattle, causing serious health troubles and financial losses [3]. Concerning developing countries, the prevalence rates of subclinical mastitis are gradually increasing, ranging from 60% to 80% [4]. On the other side, concerning developed countries, Cheng and Han [5] reported a mastitis percentage of 40% in Europe per year, costing 124 EUR annually.

Despite being the third global foodborne illness, according to Ahmed et al. [6], *S. aureus* is still a neglected and non-notifiable zooanthroponotic disease in Egypt. One-third of people become infected mainly via damaged skin and mucosa, resulting in skin infections, respiratory manifestations, septicemia, abscesses, and even death [7].

Due to the global spread of resistant bacterial strains that can swiftly evolve and disseminate resistance to subsequent strains, antibiotics are no longer frequently utilized as growth promoters. As a result, herbal bioactive compounds (phytobiotics) such as flavonoids, tannins, saponins, phenols, and essential oils (EOs) have recently become popular alternatives because they are naturally occurring, have low to no toxicity, and have a variety of other beneficial properties when used as feed additives, enhancing animals’ productivity and combating zoonotic diseases [8].

In terms of their tremendous nutritional and economic value, they are used in the agricultural and food industries as preservatives, anti-inflammatory agents, hypoglycemic antioxidants, anti-hyperlipidemics, and antimicrobials. Thyme oils (the primary source of thymol) *(Thymus vulgaris*), tea (*Melaleuca alternifolia*) oil, cactus fruit seed oil (*Opuntiaficus indica*), and the geranium essential oil, which is extracted from the aromatic leaves of the rose-scented geranium, were claimed to be superior to other herbal EOs in several studies [9–12].

There are few and very restricted Egyptian data on antimicrobial-resistant *S. aureus *strains in dairy and human samples. Therefore, it is with great pleasure that a group of Egyptian researchers conducted the current study, which was the first Egyptian research performed to determine the sensitivity of multidrug-resistant (MDR) isolates of both raw dairy cattle and human samples to these four readily available, inexpensive, non-toxic essential oils (EOs: thyme, tea, geranium, and cactus oil) in comparison with their sensitivity to other different known commercial antibiotics. This study was conducted in light of the previously mentioned antimicrobial, immunostimulant, and antioxidant activities of the preceding four EOs, as well as the poor efficacy of various known antibiotics against *S. aureus* due to a low cure rate and a great deal of consumer concern about the emergence of antibiotic resistance.

## Material and Methods

### Ethical approval

The National Research Committee of Egypt’s ethical guidelines (no. VUSC-032-1-23) were followed in all the procedures used in this investigation, including the collection of dairy and human sputum samples. Every human subject provided permission for the sputum samples to be collected provided that any information that may be used to identify them would be kept private.

### Samples collection and processing

The present study concerned two sides: first, the animal samples: 45 raw cattle milk samples (30 ml each, collected in sterile plastic tubes after teat disinfection and discarding the first few milliliters of foremilk) were collected, as per the methods described previously [13, 14], and 35 Karish cheese (unpasteurized white, soft home-made cheese) samples were collected in sterile plastic containers (100 gm each) from farmers’ houses from different districts in Giza governorate, Egypt. Second, the human samples—a total of 30 sputum samples (in sterile closed swaps)—were collected from farmers in contact with the aforementioned collected animal samples in the same governorate and showed respiratory manifestations with no response to traditional antibiotics. All the samples were gathered from June to December 2022 according to the regulations of the Institutional Animal Care and Use Committee (IACUC) under the ethical approval number VUSC-032-1-23. The samples were immediately transferred in an icebox (2–5°C) to the Zoonoses Laboratory at the National Research Center (NRC), Giza Governorate, Egypt, for further examinations.

### Isolation of Staphylococcus aureus

Plates of Baird-Parker agar (Oxoid, Basingstoke, UK) were evenly distributed with the proper dilutions of human and cheese samples, while the original milk samples were used without dilution and incubated at 37°C for 24-48 h, according to Abdurabbah et al. [15]. Shiny black colonies with a surrounding clear zone were considered positive for *S. aureus*, coagulase testing using rabbit plasma, catalase testing using 3% hydrogen peroxide, and Gram staining (Becton Dickinson and Co.). Then isolates were kept in Luria broth cultures (LB; Becton Dickinson and Co.) at 80°C with 20% glycerol.

### Antimicrobial susceptibility testing (AMS)

Various kinds of known commercial antimicrobials in the veterinary profession were utilized to check the antimicrobial susceptibility (AMS) of obtained *S. aureus* isolates: amoxicillin-clavulanic (30µg), clindamycin (2 µg), cefoxitin (30 µg), doxycycline (30 µg), sulfamethoxazole-trimethoprim (30 µg), cefotaxime (30 µg), ampicillin (10 µg), ceptaxime (30 µg), erythromycin (15 µg), ciprofloxacin (5 µg), and imipenem (10 µg). According to Abdeen et al. [16], disc diffusion experiments were performed (in triplicate) on plates of Muller Hinton agar (Oxoid, Ltd., Basingstoke, Hampshire, UK) with the observation of any inhibition zones where multidrug resistance (MDR) is the capacity of *S. aureus* isolates to survive two or more antimicrobials from distinct classes.

### Molecular detection of S. aureus using conventional Polymerase Chain Reaction (PCR)

To identify *S. aureus* (*nuc* gene) and MRSA (*mec*A gene) with a further screening of all the obtained isolates for Vancomycin resistance using multiplex PCR targeting *van*A, *van*B, and *van*C genes, DNA was extracted from positive Baird-Parker samples and processed according to QIAamp DNA mini kit instructions (Qiagen, Germany, GmbH) by Gharaibeh et al. [17]. The GS-96 gradient thermocycler (Hercuvan, Malaysia) was used to conduct PCR reactions according to Abdeen et al. [16], with a final reaction volume of 25 µl. Ultimately, the obtained DNA was separated on a 1.5% agarose gel by electrophoresis, and Syngene’s InGenius3 gel documentation system was used for gel analysis. The current study utilized the primers listed in Table 1.

### Agar well diffusion method

Similar to the previously reported disk-diffusion methodology, the agar-well diffusion method was widely employed to assess the antibacterial activity of different plant extracts [22]. A volume of the microbial inoculum was evenly spread over the whole surface of the agar plates. Agar plates were left to be incubated at 32°C for 24 h after aseptically drilling holes with a diameter of 6 to 8 mm and adding the appropriate concentration of EOs extract solutions in a volume of 50–100 µl into the holes. The zones of inhibition were identified following incubation.

### Statistical analysis

According to Byomi et al. [23], data analysis was conducted using Statistical Package for Social Science (SPSS) version 17. Furthermore, percentages were calculated to represent the frequency of contamination.

## Results

### Cultural method for the isolation of S. aureus

Table 2 declares that out of 110 samples tested, 22 isolates were exactly declared as *S. aureus,* with an incidence of 20%. The highest incidence was 28.57% (10 out of 35) in Karish cheese, followed by sputum samples at 20% (6 out of 30) and raw cattle milk at 13.33% (6 out of 45).

**Table 1.** PCR primers and probes used in the current study.

Gene	Sequence (5′-3′)	Amplicon size (bp)	Reference
*nuc*	CTGGCATATGTATGGCAATTGTT TATTGACCTGAATCAGCGTTGTCT	664	[17]
*mec*A	GTAGAAATGACTGAACGTCCGATAA CCAATTCCACATTGTTTCGGTCTAA	310	[18]
*van*A	GGGAAAACGACAATTGC GTACAATGCGGCCGTTA	732	[19]
*van*B	ATGGGAAGCCGATAGTC GATTTCGTTCCTCGACC	635	[20]
*van*C	CTCCTACGATTCTCTTG CGAGCAAGACCTTTAAG	439	[21]

### Results of antimicrobial susceptibility testing

Tables 3 and 4 represent that the dairy and human samples were resistant to amoxicillin-clavulanic and ampicillin antibiotics, whereas the dairy samples showed further resistance against ceptaxime and an intermediate reaction against erythromycin.

**Figure 1.**
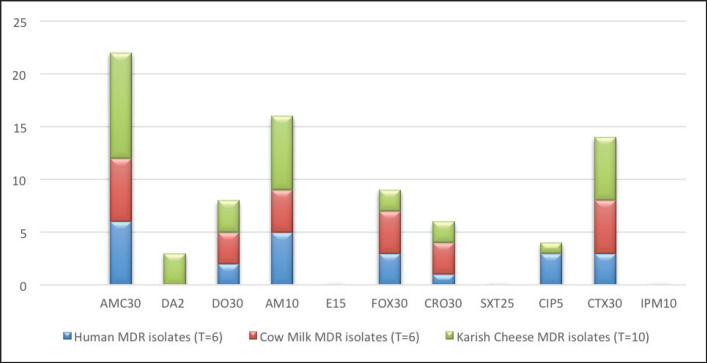
Antibiotic resistance pattern along tested isolates from different origins using Agar disc diffusion test (ADDT).

**Figure 2.**
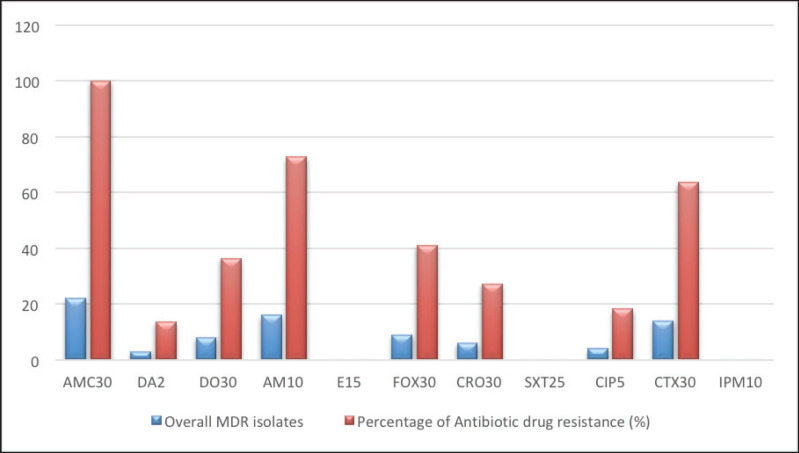
Overall antibiotic resistance pattern among tested isolates.

Whereas Figures 1 and 2 show that 14 out of 22 *S. aureus* isolates showed multiple drug resistance (MDR).

### Results of polymerase chain reaction (PCR)

Out of 22 positive *S. aureus* isolates detected by isolation on Baird-Parker media, only 14 represented multidrug resistance (MDR) (as detected by antimicrobial susceptibility testing). These 14 isolates were used for confirmation by conventional and multiplex PCR. Those 14 positive PCR samples were (6 raw cattle milk, 5 Karish cheese, and 3 human samples), as identified by using conventional PCR.

**Figure 3.**
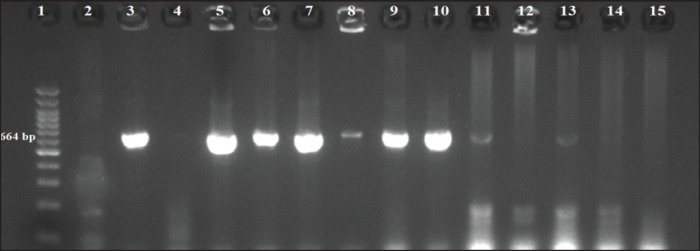
PCR-amplified *S. aureus nuc* gene (664 bp) product on agarose gel electrophoresis. Lane 1: 100 bp DNA Ladder; Lanes 3, 5, 6, and 7: positive cow milk samples, 8, 9, and 10: positive Karish cheese samples, 11, and 13: positive human samples, lanes 2, 4, 12, 14, and 15: negative results.

**Figure 4.**
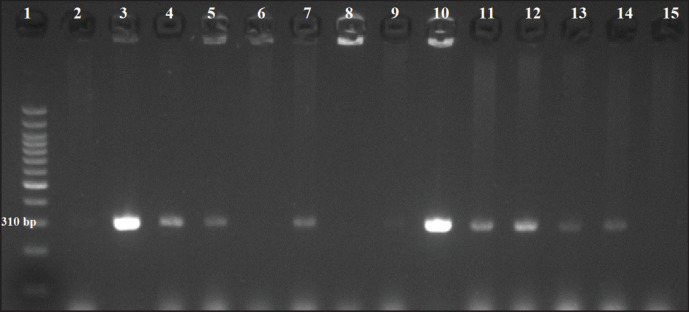
MRSA *mec*A gene-amplified PCR product (310 bp) on agarose gel electrophoresis. Lane 1: 100 bp DNA ladder; Lanes 3, 4, and 5: positive cow milk samples, 7, 10, 11, and 12: positive Karish cheese samples, 13, and 14: positive human samples, lanes 2, 6, 8, 9, and 15: negative results.

**Figure 5.**
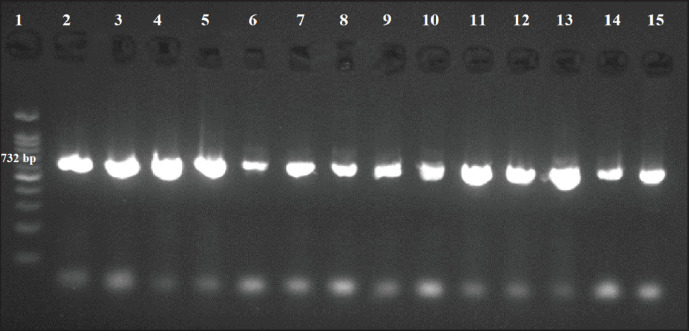
PCR-amplified *S. aureus van*A gene on agarose gel electrophoresis. *van*A gene (732 bp), *van*B gene (635 bp), *van*C gene (439 bp). Lane 1: 100 bp DNA ladder; Lanes, 2-15: showing only positive *van*A gene results.

Furthermore, by application of multiplex PCR, Figure 3 represents a positive *nuc* gene that was detected in 4 out of 6 (66.6%) cow milk samples, 3 out of 5 (60%) Karish cheese samples, and 2 out of 3 (66.6%) human samples. Figure 4 shows the results of the *mec*A gene that was positive in 3 out of 6 (50%) cow milk samples, 4 out of 5 (80%) Karish cheese samples, and 2 out of 3 (66.6%) human samples. Figure 5 declares that all 14 positive PCRs (obtained by conventional PCR) were positive only for *the van*A gene (no *van*B nor *van*C genes were recorded).

### Agar-well diffusion testing by using different concentrations of tested oil extracts on selected MRSA isolates

**Figure 6.**
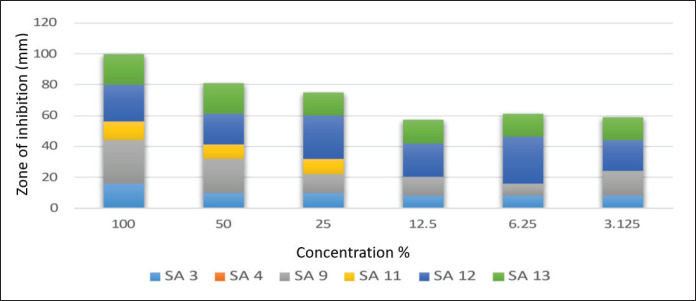
Mean inhibitory zone of Cactus oil extract against tested MRSA isolates.

**Figure 7.**
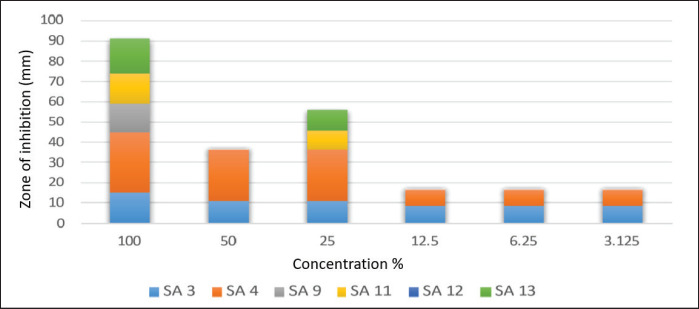
Mean inhibitory zone of Geranium oil extract against tested MRSA isolates.

Results in Figure 6 indicate that the concentrated form of cactus oil (100%) shows the best inhibitory activity against all tested MRSA isolates, followed by conc. 50%, then 25%. Results indicate that the concentrated form of cactus oil (100%) shows the best inhibitory activity against all tested MRSA isolates. Whereas Figure 7 showed that SA3 and SA4 were affected by geranium oil extract at different concentrations, other tested MRSA isolates were not inhibited by the diluted form of geranium oil extract.

**Table 2.** Isolation of *S. aureus* using Baird-Parker agar from different sources.

Test	Total no. of examined samples	Raw cattle milk samples		Karish cheese		Human (sputum) samples		Total positive samples	
		Total no. = (45)		Total no. = (35)		Total no. = (30)			
		Positive no.	Positive %	Positive no.	Positive %	Positive no.	Positive %	No.	%
Isolation of *S. aureus* on Baird-Parker agar plates	(110)	6	13.33	10	28.57	6	20	22	20

**Table 3.** Antimicrobial susceptibility test of MRSA isolates from dairy samples (raw milk and Karish cheese).

Antimicrobial agent	Conc. (µg)	R	I	S
Amoxicillin-clavulanic (AMC)	30	100	0	0
Clindamycin (DA-20)	2	18.75	31.25	50
Doxycycline (DO30)	30	31.25	12.5	56.25
Ampicillin (AM 10)	10	68.75	6.25	25
Erythromycin (E 15)	15	0	87.5	12.5
Cefoxitin (FOX30)	30	50	0	50
Cefotaxime (CRO)	30	0.25	6.25	68.75
Sulfamethoxazole-trimethoprim (SXT25)	30	0	6.25	93.75
Ciprofloxacin (CIP 5)	5	6.25	43.75	56.25
Ceptaxime (CTX30)	30	62.5	0	37.5
Imipenem (IPM10)	10	0	0	100

R: Resistant %, I: Intermediate %, S: sensitive %

**Figure 8.**
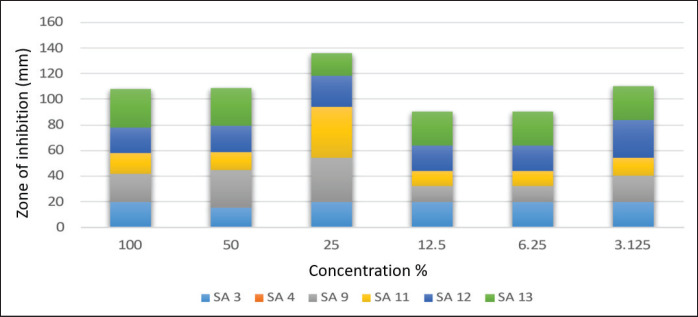
Mean inhibitory zone of Tea oil extract against tested MRSA isolates.

Results in Figure 8 indicate that the lower concentration of tea oil (25%) shows the best inhibitory activity against all tested MRSA isolates. All MRSA isolates were affected by tea oil extract at different concentrations, except isolate SA4, which was not inhibited by tea oil extract at any concentration.

**Figure 9.**
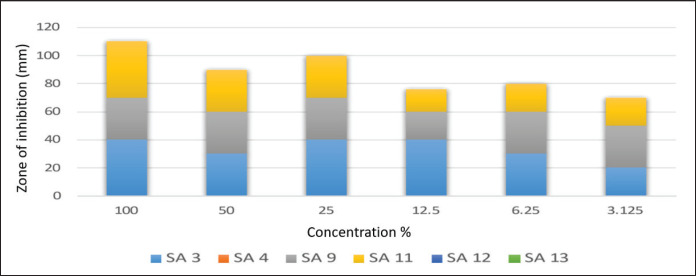
Mean inhibitory zone of Thyme oil extract against tested MRSA isolates.

Results of Figure 9 indicate that the concentrated form of thyme oil (100%) showed the best inhibitory activity against three MRSA isolates (SA3, SA9, and SA11). The isolates are inhibited at different dilutions of thyme oil. On the other hand, some MRSA isolates show complete resistance against thyme oil extract at all concentrations (SA4, SA12, and SA13).

## Discussion

In the current investigation, S. aureus isolation from dairy and human samples revealed that 13.33% of milk samples, 28.57% of Karish cheese samples, and 20% of human samples were positive, for a total of 20%. The obtained result of the milk samples agreed with a percentage of 12.74% in India [24]. Higher percentages of 41.1% and 44% were previously mentioned in Egypt in Dakahlia and Menoufia governorates, respectively [3, 16]. In other parts of the world, a higher isolation rate of 24.9% was detected in Ethiopia [25], while another lower isolation percentage of 3.9% was detected in China [26].

Regarding Karish cheese, a previous Egyptian study in Dakahlia governorate revealed a lower isolation percentage of 16.7% [3]. However, a higher previous Egyptian isolation rate was declared at 44% in the Menoufia governorate [16]. Another high isolation rate was reported as 70% (21 out of 30) in Iran [27]. Regarding the human result, it was somewhat in agreement with the isolation rate of 25% in Brazil [28].

Concerning the obtained antibiotic susceptibility testing results, all the isolated S. aureus showed resistance against amoxicillin-clavulanic (100%) and ampicillin antibiotics (75% and 83.3% among dairy and human samples, respectively). In addition, further resistance and intermediate reactions were detected against ceptaxime (62.5%) and erythromycin antibiotic (87.5%) among dairy samples, respectively.

The current results agree with Abdeen et al. [29], who represented through their Egyptian study in Qaluobyia governorate the resistance of S. aureus isolates against erythromycin (52.8%) and amoxicillin (91.4%) among dairy and human samples. On the contrary, Abdeen et al. [16] showed the susceptibility of S. aureus isolates to amoxicillin/clavulanate (46.9%) in Menoufia governorate, Egypt. According to Gebremedhin et al. [30]**,** ampicillin resistance was most prevalent in Central Ethiopia.

In 14 isolates exhibiting strong drug resistance (MDR), nuc and mecA gene presence was assessed using conventional PCR. Results showed that 66.67% of human samples, 60% of Irish cheese samples, and 66.67% of raw cattle milk samples contained the nuc gene (a unique marker gene), which concurs with the findings of Sahebnasagh et al. [31], who noted that some phenotypically S. aureus isolates displayed negative results, most likely as a result of subpar experimental conditions for the PCR method, because of various mutations, or because some S. aureus strains lack the nuc gene. In light of this, it only makes sense that S. aureus cannot be completely excluded from clinical isolates based only on the results of a negative PCR test for the nuc gene.

Further molecular analysis was done to find the mecA gene (a helpful molecular indicator of methicillin resistance) in the tested isolates, and the results showed that it was present in 66.67% of human samples, 80% of Karish cheese samples, and 50% of raw cattle milk samples.

Following the discovery of vancomycin-resistant S. aureus strains (VRSA) in Japan in 1996, researchers notified these strains on other continents. Africa exhibited the highest prevalence of both VRSA and VISA (vancomycin intermediate-resistant S. aureus), followed by Asia, South America, North America, and Europe [32]. The main reasons for the higher prevalence of VRSA in Africa than in wealthy countries include subpar hygiene standards, poor nosocomial infection surveillance, and incorrect use of the available antibacterial drugs, with further transmission of these strains to animals in contact [32].

In the current investigation, multiplex PCR (mPCR) declared all 14 PCR-positive isolates were positive for vanA genes, but none of them were positive for vanB or vanC genes. Only 19.3% of S. aureus isolates were identified to have the vanA gene in a previous Egyptian investigation conducted by Muzammil et al. [33]. Furthermore, in Pakistan, Vazquez-Rosas et al. [34] reported that 10.89% of bovine milk samples were genotypically positive for VRSA. On the other side, previous research failed to find any vanA gene in all the tested human samples [35], whereas in the United States, Ezzat et al. [36] reported that 71% of examined human cases were positive for VRSA infection.

The results obtained in this investigation were relatively comparable to those of Metersky et al. [37] and Toubar et al. [38], who reported PCR-positive rates of 11.3% and 16% in milk and human sputum samples, respectively, in the Port Said governorate, Egypt, and the United States, respectively. Conversely, in the governorate of Port Said, Egypt, La Storia et al. [39] reported a lower PCR proportion of 8% in Karish cheese.

The obtained results in the present research are still lower than the PCR positivity obtained by other researchers, such as 60% in Karish cheese in Dakahlia governorate, Egypt [3]. Moreover, PCR genes were detected with an incidence of 42.5% in human samples in Argentina [40].

In clinical and veterinary settings, interest in the potential of herbal medicine has increased [10]. Gram-positive bacteria tend to be more resistant than Gram-negative bacteria since their outer membrane is less permeable. Despite this, the studies that have been done on the impact of EOs on S. aureus are still underrated. Regarding the obtained results of agar well diffusion testing, the current study declared that the cactus oil extract was the best-used extract for all tested MRSA isolates, followed by the tea oil extract that showed the best hindrance activity among all tested MRSA, especially at 25% concentration. The third effective essential oil extract was the thyme oil extract, where the highest hindrance activity was noticed against SA3, SA9, and SA11 only. The last oil extract was the geranium oil extract, which had antimicrobial activity against SA12 and SA13 at concentrations of 100 and 25%, respectively.

On the other hand, Ramírez-Moreno et al. [41] failed to find any discernible antimicrobial affectivity of cactus oil extract against S. aureus. Shi et al. [42] concluded through their in vitro studies that tea tree oil had a strong microbicidal action against S. aureus (in biofilms or even in suspensions) at relatively low doses. Additionally, an applied study by Vázquez-Sánchez et al. [43] on MRSA patients discovered that a combination of a body wash containing 5% tea tree oil and a nasal ointment containing 4% tea tree oil was more effective in eradicating MRSA carriage than a standard body wash containing triclosan and a 2% nasal ointment containing piprovincin.

**Table 4.** Antimicrobial susceptibility testing in MRSA isolates from human (sputum) origin.

Antimicrobial agent	Conc. (µg)	R%	I%	S%
Amoxicillin-clavulanic (AMC)	30	100	0	0
Clindamycin (DA-20)	2	0	33.3	66.6
Doxycycline (DO30)	30	33.3	0	66.6
Ampicillin (AM 10)	10	83.3	0	16.6
Erythromycin (E 15)	15	0	50	50
Cefoxitin (FOX30)	30	50	0	50
Cefotaxime (CRO)	30	16.6	33.3	50
Sulfamethoxazole-trimethoprim (SXT25)	30	0	16.6	83.3
Ciprofloxacin (CIP 5)	5	50	0	50
Ceptaxime (CTX30)	30	50	0	50
Imipenem (IPM10)	10	0	0	100

R: Resistant %, I: Intermediate %, S: sensitive %.

Furthermore, it was shown that thyme oil in sub-lethal quantities suppresses the growth of planktonic cells, which is comparable to how it slows down S. aureus development due to its high availability of thymol and carvacrol, which disrupt numerous metabolic processes and gene responses, deplete intracellular adenosine triphosphate (ATP), block ATPase, and change the permeability of membranes [44]. Thymol also prevents StaphEnterotoxins A and B (SEA and SEB) and α-hemolysin from being produced and secreted by S. aureus [45].

Furthermore, Kwieciński et al. [46] concurred with the current results of the geranium oil extract, indicating that, in comparison to other EOs, geranium oil was the least efficacious EO for treating S. aureus. The current study’s findings showed clear differences in the rankings of EOs based on how effective they were against S. aureus as an antibacterial agent. Thus, it seems that factors such as the rate of diffusion and hydrophobicity of EOs may significantly affect their effectiveness.

## Conclusion

Antibiotic resistance has prompted researchers to look for new, natural substances that have antibacterial properties. The results of this study are very encouraging because they provide credence to the theory that some essential oils, like those from cacti, tea, thyme, and geranium plants, have a potent antimicrobial effect on zoonotic *S. aureus* and could serve as natural antibacterial agents in Egypt to lessen the overuse of antibiotics in veterinary and medical settings.

## List of Abbreviations

°C, degree centegrate; µg, microgram; µl, microliter; AMS, antimcrobial susceptibility; DNA, deoxy ribonucleic acid; Eos, essential oils; IACUC, Institutional Animal Care and USA Committee; MDR, multi drug resistance; mg, milligram; mm, millimeter; MRSA, methicillin resistant *Staphylococcus aureus; *NRC, National Research Center; PCR, polymerase chain reaction; *S. aureus*, *Staphylococcus aureus; *SPSS, statistical package for social science.
